# TAGADA: a scalable pipeline to improve genome annotations with RNA-seq data

**DOI:** 10.1093/nargab/lqad089

**Published:** 2023-10-16

**Authors:** Cyril Kurylo, Cervin Guyomar, Sylvain Foissac, Sarah Djebali

**Affiliations:** GenPhySE, Université de Toulouse, INRAE, INPT, ENVT, Toulouse, France; GenPhySE, Université de Toulouse, INRAE, INPT, ENVT, Toulouse, France; GenPhySE, Université de Toulouse, INRAE, INPT, ENVT, Toulouse, France; IRSD, Université de Toulouse, INSERM, INRAE, ENVT, Univ Toulouse III - Paul Sabatier (UPS), Toulouse, France

## Abstract

Genome annotation plays a crucial role in providing comprehensive catalog of genes and transcripts for a particular species. As research projects generate new transcriptome data worldwide, integrating this information into existing annotations becomes essential. However, most bioinformatics pipelines are limited in their ability to effectively and consistently update annotations using new RNA-seq data. Here we introduce TAGADA, an RNA-seq pipeline for Transcripts And Genes Assembly, Deconvolution, and Analysis. Given a genomic sequence, a reference annotation and RNA-seq reads, TAGADA enhances existing gene models by generating an improved annotation. It also computes expression values for both the reference and novel annotation, identifies long non-coding transcripts (lncRNAs), and provides a comprehensive quality control report. Developed using Nextflow DSL2, TAGADA offers user-friendly functionalities and ensures reproducibility across different computing platforms through its containerized environment. In this study, we demonstrate the efficacy of TAGADA using RNA-seq data from the GENE-SWiTCH project alongside chicken and pig genome annotations as references. Results indicate that TAGADA can substantially increase the number of annotated transcripts by approximately $300\%$ in these species. Furthermore, we illustrate how TAGADA can integrate Illumina NovaSeq short reads with PacBio Iso-Seq long reads, showcasing its versatility. TAGADA is available at github.com/FAANG/analysis-TAGADA.

## Introduction

Since the publication of the first animal genome more than two decades ago, genome sequencing has become easier, faster and cheaper by several orders of magnitude. Genome annotation, however, did not benefit from the same progress. Annotating a given genomic sequence aims to identify the nature and position of its encoded functional elements, most important of which are genes and transcripts. In this context, transcriptome sequencing experiments like RNA-seq can provide useful information in order to describe in a resulting gene annotation –hereafter called annotation– the precise exon–intron structure of each transcript present in a biological sample of interest. To capture most of the transcriptome diversity that can potentially be produced by a species of interest, genome annotation projects typically generate and process RNA-seq data from several samples of diverse biological origins, including different tissues, organisms, and conditions. The task is challenging and highly demanding in terms of computational resources.

Consequently, genome annotations are usually performed by large groups of experts. Naturally, given their prime access to the raw data and their analysis resources, genome sequencing consortia are usually providing genome sequences with their corresponding reference annotations (1,2). Dedicated groups have been specializing in genome annotation, either with a species-specific focus (3–6) or in a more generic and systematic spectrum (7–9). These groups provide new annotations on a regular basis, in particular when a new genome assembly version or a large transcriptomic dataset is made available. Resulting gene models are then used and referred to as reference annotations by the community. They highly depend on diverse criteria (species of interest, type and amount of processed information, annotation source, etc.) that can impact their quality and the one of subsequent studies (10–12). Naturally, they also reflect the knowledge status of the transcriptomic field. Long non-coding RNAs (lncRNAs) for instance used to be often discarded from reference annotations. They now account for most of the annotated genes in human and mouse. Genome annotation also follows technological progress. While most of the transcriptomic projects still heavily rely on Illumina short-read RNA-seq data, long-read sequencing technologies are being increasingly used, allowing for full-length transcript identification.

An obvious challenge to maintain a reference annotation up to date is to keep up with the growing rate of new sequencing data being produced world-wide. The unavoidable delay between the availability of transcriptomic datasets and their integration into reference annotations urges the need for research groups to update reference annotations on their own, in particular when investigating transcriptomes of hitherto uncharacterized tissues, developmental stages or biological conditions. Many software tools allow to build a *de novo* new gene annotation from transcriptomic data, either using a reference genome (13) or not (14), and many pipelines have been developed to perform RNA-seq data analysis (15–22). Nevertheless, few of them address the typical use case of building upon an existing annotation to augment it instead of replacing it. In particular, combining technical replicates—e.g. sequencing runs from the same library—, biological replicates—e.g. from the same tissue or under the same experimental conditions—and an existing reference is not often addressed by existing RNA-seq bioinformatics pipelines.

In addition, with the growing need for standardization in the field of computational data analysis, containerized pipelines are becoming widely adopted to ensure reproducible results regardless of the computational environment. The emergence of the nf-core pipeline collection (19) illustrates this trend. The nf-core/rnaseq pipeline for instance allows to quantify gene and transcript expression from a reference annotation, and, more recently, to assemble new annotations from individual samples. As it was not specifically designed for gene annotation, it does neither address the merging process across samples nor the downstream characterization of the resulting genes and transcripts, e.g. lncRNA detection.

To summarize, existing RNA-seq data analysis pipelines suffer from at least one of the following limitations:

- Dependency on the local software environment, resulting in a lack of reproducibility
- No possibility to generate a novel annotation. In the best case of the nf-core/rnaseq pipeline, different novel annotations are generated but no integration into a single annotation is proposed. Consequently, only the reference annotation can be used to compare expression levels between conditions
- No lncRNA detection/characterization

Supplementary Table S1 provides a descriptive list of currently available RNA-seq pipelines.

Here, we present TAGADA (Transcripts And Genes Assembly, Deconvolution, Analysis), a containerized pipeline to update genome annotations using RNA-seq data. Given a genomic sequence, a reference annotation and a set of read files, TAGADA builds upon the reference to generate a novel annotation. It also quantifies genes and transcripts’ expressions from both the reference and the new annotation. Compared with traditional RNA-seq analysis tools, TAGADA performs a complete end-to-end annotation of the target genome, combining results from a large set of samples to deliver a final GTF file. It automatically performs numerous Quality Controls, and identifies long non-coding transcripts from the new annotation using a dedicated FEELnc module. TAGADA is also versatile, as it can directly process previously generated BAM files, and integrate ‘short-read’ data into a ‘long-read’ annotation from PacBio or Oxford Nanopore technologies. By processing a large RNA-seq dataset from two animal species (chicken and pig), we show here that TAGADA can successfully integrate transcriptomic data to enhance a reference annotation in a reliable way.

## Materials and methods

### The TAGADA pipeline

#### Pipeline overview

**Figure 1.**
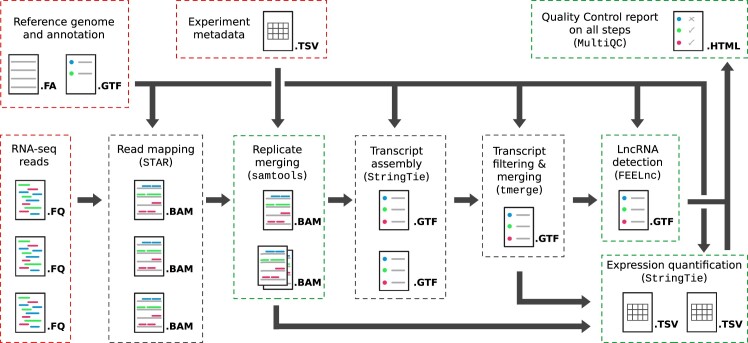
Overview of the TAGADA pipeline. TAGADA takes as input a reference genome and a reference gene annotation, RNA-seq reads and optionally experiment metadata (framed in red boxes). Its main steps are framed in grey or green boxes depending on whether the step produces intermediate or final pipeline outputs, and include the step name, the underlying tool and the format of the output files produced.

TAGADA is a pipeline for the structural annotation and expression quantification of genes and transcripts. An overview of the main workflow is presented in Figure 1. In brief, TAGADA takes as input a set of RNA-seq read files, a genomic sequence, a reference annotation, and an optional metadata file listing attributes of the provided RNA-seq data (e.g. tissue, condition, age, gender, etc). After trimming the reads with Trim Galore (https://www.bioinformatics.babraham.ac.uk/projects/trim_galore/), TAGADA maps them to the genome and to the annotated exon-exon junctions using STAR (23), and optionally merges read alignments from samples with common attributes using samtools (24). It then assembles the resulting read mappings into transcripts using StringTie (25), and merges all the obtained assemblies with the reference annotation into a novel gene annotation using tmerge (https://github.com/guigolab/tmerge). Various filtering steps are applied to discard unreliable transcripts based on expression or consistency across samples (see ‘Transcript filtering’ below). The coding status of the annotated transcripts is then assessed in order to identify lncRNAs in the novel annotation using the FEELnc lncRNA predictor (26). A resulting annotation file is delivered in the standard GTF format. In addition, TAGADA quantifies the expression of transcripts and genes from the reference and the novel annotation using StringTie (25), and provides expression tables for both annotations separately. Expression values are provided for each gene and transcript across samples in the form of raw read counts and TPM-normalized values (Figure 1).

#### Key features

##### An annotation-focused pipeline

TAGADA’s main goal is to improve a reference annotation by finding new genes and transcripts. As a consequence, an existing annotation file has to be provided. It might get extended with additional genes and transcripts, but reference elements cannot be removed and are systematically reported into the novel annotation without modification, except in case of 5’ and/or 3’ exon extension. The identication of a novel transcript isoform of an existing gene might for instance require updating the gene boundaries. Likewise, a reference transcript that has been extended at an extremity is discarded in favour of the longer novel isoform (see the following section).

##### Merging transcriptome data and results

Another consequence of this annotation approach is the need to encompass all the transcriptome diversity of the input samples in a unique annotation file, as in the provided reference annotation. Since sequencing reads are produced from samples of various biological sources, this implies merging the data at some step of the workflow.

When, what, and how to merge are important yet often overlooked questions (27). Different levels and methods have to be considered. At the read level for instance, it is correct to combine all runs from the same sequencing library. TAGADA can therefore handle such technical replicates when they are provided as several FASTQ files. One might not want, on the other hand, to combine reads that come from all the samples to assemble transcripts, especially when samples come from different tissues or experimental conditions. Consequently, TAGADA allows to define which samples should be merged together or kept separately during transcript assembly (assemble-by option).

The resulting transcriptomes still need to be merged into one single annotation file. Redundancy across the corresponding GTF files makes a simple concatenation not satisfying, as it would result in many repeated transcripts. The process, however, should avoid the creation of virtual chimera that are absent from the individual samples. In this regard, TAGADA adopts a conservative approach: overlapping transcripts can be collapsed together only if they have exactly the same intron chain, which restricts possible modifications to extending the first or last exonic position. Transcripts that are strictly included in others are simply discarded, as they do not provide additional information. At the gene level, a more permissive approach is chosen: transcripts that share a common exonic position on the same strand are given the same gene ID.

Additional characterizations of TAGADA transcripts and genes are automatically conducted using the reference annotation. If a TAGADA transcript’s exon structure exactly matches that of a reference transcript, it is assigned the reference transcript ID (transcript_id) and corresponding biotype (transcript_biotype). Likewise, if a TAGADA gene has an exon that overlaps that of a reference gene, a ref_gene_id attribute listing the corresponding gene ID(s) is defined.

##### Transcript filtering

Technical artifacts in several steps of the process, including library preparation, read mapping and transcript assembly, make each transcriptome to be merged susceptible to include a variable degree of noise, that can then contaminate the final annotation. Therefore the absence of filtering steps would yield an artificially inflated number of transcripts in the final annotation, which would be polluted with several spurious models. This is a serious issue, in particular for large scale transcriptomic projects that involve a high number of samples. To prevent this typical noise inflation, TAGADA adopts two transcript filtering steps:

- a consistency-based filter: novel transcripts that are detected in less than a user-defined number of transcriptomes (2 by default) are discarded from the final annotation. This allows to remove random transcriptional noise while keeping condition-specific transcripts that are rarely yet consistently observed,
- an expression-based filter: novel transcripts for which the highest TPM expression level across all transcriptomes does not reach a user-defined threshold (0.1 by default) are also discarded. The TPM expression value that is considered for this filtering step is generated by StringTie during the previous ”Transcript assembly” step of the pipeline (Figure 1).

##### Quantification of expression

As for the transcriptome assembly step, input data can be aggregated at a custom level to quantify gene and transcript expression. Unlike the nf-core/rnaseq pipeline, TAGADA does not require the quantification to be performed at the same step as the transcriptome construction. This can be helpful in the typical use case of differential gene expression analysis: while the transcriptome is characterized at the level of a specific factor like tissue or experimental condition, gene expression needs to be assessed for each biological replicate of that factor. In addition, a hierarchical clustering of the input samples is automatically performed using the reference expression profiles and the optional experimental metadata file.

##### Long non-coding RNA detection

As part of the TAGADA pipeline, FEELnc (26) assigns a specific feelnc_biotype attribute (lncRNA, mRNA, TUCp for Transcripts of Unknown Coding potential, noORF) to a fraction of TAGADA transcripts. Indeed to be considered by FEELnc, a transcript needs to be longer than 200 bp, to have no exonic overlap with a reference mRNA transcript on the same strand, and to have either strictly more than two exons or exactly two exons that are both longer than 25 bp. Detected lncRNAs are further characterized depending on their relative position with respect to their closest coding transcript, including configurations such as genic exonic or intronic, intergenic downstream or upstream (sense and antisense).

##### Flexibility and usability

Additional features of the pipeline include:

- multiple entry points to start from raw sequenced reads in FASTQ format or from mapped reads in BAM format,
- automatic detection of directional sequencing,
- extensive QC charts and reports, including a comparison of the novel vs. reference annotation: number of identical transcripts, alternative isoforms, extensions, etc.

##### Implementation

TAGADA is written in Nextflow DSL2 (28) and includes scripts from various languages (Python, bash, etc). The Nextflow framework is specifically designed to develop reproducible, scalable and portable analysis workflows (19) Consequently, TAGADA can run on various computing platforms and is available in the form of a Docker or Singularity container. It has been used on a Kubernetes cloud, on local computers as well as on SGE and SLURM clusters. The code is open-source and available at https://github.com/FAANG/analysis-TAGADA.

### Use case: transcriptome profiling in chicken and pig

#### Context and biological material

TAGADA was initially developed and tested to improve the chicken and pig genome annotations in the context of the GENE-SWitCH project (regulatory GENomE of SWine and CHicken: Functional Annotation during development, https://www.gene-switch.eu/). As part of the FAANG initiative ((29), www.faang.org), the GENE-SWitCH project is a European project aiming at improving the functional annotation of the chicken and pig genomes during development, with a focus on gene expression regulation, and with the final goal to improve animal breeding. For the functional annotation of these species, a panel of tissues from four animals (two males and two females) from three developmental stages of each species were sampled, and seven core tissues were used to improve the reference genome annotation (Table 1). For each species, these 84 samples (7 tissues, 3 developmental stages, 4 animals) were interrogated using various experimental assays, including Illumina and PacBio RNA-seq technologies for transcriptome profiling.

**Table 1.** Experimental design of the RNA-seq data from the GENE-SWitCH project used in this study

2 species	Chicken (*Gallus gallus*)
	Pig (*Sus scrofa*)
4 replicates	2 males
	2 females
3 developmental stages	Early organogenesis
	Late organogenesis
	Hatched/New born
7 core tissues	Brain, intestine, kidney, liver, lung, muscle, skin

A total of 168 pairs of FASTQ files have been processed, which corresponds to 84 samples per species.

#### RNA-seq reads

Illumina RNA-seq reads were obtained from ENA under accession IDs PRJEB42025 (chicken) and PRJEB41970 (pig). Briefly, RNA-seq paired-end libraries (2 × 150 bp) were generated from polyA+ enriched RNAs using a directional and high-depth sequencing protocol. About 100 million read pairs were obtained per library using an Illumina NovaSeq 6000 (see metadata files in supplementary data file 1). PacBio long reads were not directly used by TAGADA but the Iso-Seq-generated annotation (ENA accession IDs ERZ15610616 and ERZ15610622) was provided to it as a reference.

#### TAGENS annotation generation

For each species, TAGADA v2.1.0 was launched on a Kubernetes cluster using a Docker container image and Nextflow v21.04.1, to process the 84 RNA-seq FASTQ files using the following parameters: genome version GRCg6a (chicken) and Sscrofa11.1 (pig), genome annotation ENSEMBL v102 (called ENSEMBL in the text), –max-cpus 7 –max-memory 31GB –assemble-by tissue,stage –quantify-by tissue,stage,replicate.

#### Sample similarity heatmap and hierarchical clustering

**Figure 2.**
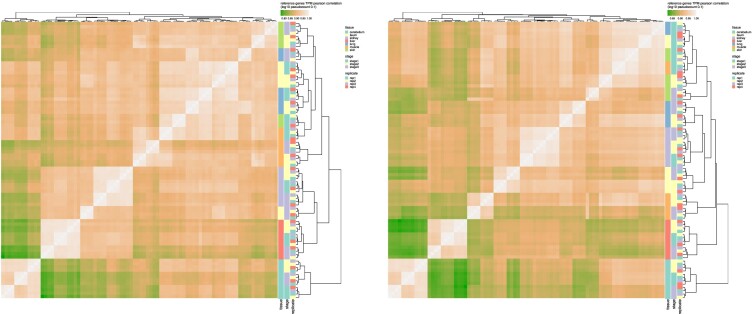
Chicken (left) and pig (right) sample similarity and hierarchical clustering produced by the TAGADA pipeline. On the heatmaps, the colour of a cell represents the similarity between the two corresponding samples (the lighter the higher), and is computed as the Pearson correlation between the log_10_ of the TPMs plus 0.1 of all reference genes. A sample hierarchical clustering is made using this similarity measure and a complete linkage aggregation method. Samples are also coloured by tissue, developmental stage and replicate.

In each species, the similarity between RNA-seq experiments/samples was computed as the Pearson correlation of the *log*10 of the TPM expression of all the reference genes (after adding a pseudo-count of 0.1, matrix_to_dist.R script). This similarity was represented as a squared heatmap of dimension the number of samples. Each sample was also coloured by three different attributes: the tissue, the developmental stage and the replicate number (1–2 for males, 3–4 for females). Finally the samples were clustered based on their similarity and a complete linkage aggregation method (ggheatmap.R script). The corresponding plots were generated by the TAGADA pipeline and slightly edited to produce Figure 2.

#### Tissue specificity

In order to identify genes specifically expressed in a given tissue, normalized TPM values of reference ENSEMBL genes were averaged for all samples of a tissue. A tau tissue specificity index was computed as defined in (30). Genes with a tau index higher than 0.9 were considered as tissue specific, and were assigned to the tissue in which they were the most highly expressed.

#### GO term enrichment analysis

Tissue specific genes and their human orthologs in ENSEMBL were linked to their respective Gene Ontology (GO) terms using the AnnotationHub Bioconductor package version 3.8.0 (https://bioconductor.org/packages/release/bioc/html/AnnotationHub.html). Functional enrichment analysis of tissue specific genes was tested using the topGO package version 2.44.0 (https://bioconductor.org/packages/release/bioc/html/topGO.html), using the weight01 algorithm and the Fischer statistics.

#### TAGENS transcript positional class with respect to the reference annotation

During the TAGADA processing, TAGENS transcripts were initially assigned to positional classes with respect to reference transcripts (comptr program). Predicted transcripts were first divided into monoexonic and spliced transcripts. Spliced transcripts were further subdivided into the five following classes, and in this order in case of conflict (Supplementary Figure S1):

- Exact if there is a reference transcript whose intron chain is exactly the same as the one of the predicted transcript,
- Extension if there is a reference transcript
  - overlapping the predicted transcript on the same strand,
  - with an identical intron chain on the overlapping portion,
  - with at least one less intron in 5′ and/or in 3′ of the predicted transcript.
- Inclusion if there is a reference transcript that is an Extension of the predicted transcript,
- Overlapping if there is a reference transcript that overlaps the predicted transcript on the same strand (but does not qualifies as Exact, Extension or Inclusion),
- Intergenic_or_Antisense otherwise.

Another transcript novelty classification, called known/unknown, was conducted in the context of this study to further characterize TAGADA’s results beyond the automatic classification. A known TAGENS transcript was simply defined as any TAGENS transcript (mono or multi-exonic) whose exon–intron structure exactly corresponded to the one of a reference transcript.

This transcript novelty classification was associated to the known/enriched/unknown gene novelty classification. A TAGENS gene was defined as known if all its transcripts were known, unknown if all its transcripts were unknown and enriched otherwise.

#### TAGENS transcript and gene coding status assignment

As an outcome of the pipeline, TAGENS transcripts were either assigned:

- both a reference and a FEELnc transcript biotype,
- only a reference transcript biotype,
- only a FEELnc transcript biotype,
- neither a reference nor a FEELnc transcript biotype.

To simplify the subsequent analyses, we combined these biotypes to generate a unified transcript biotype for each TAGENS transcript, giving priority to mRNA/protein_coding biotypes in case of conflict. We also further assigned a coding biotype to TAGENS genes as follows:

- if a TAGENS gene includes at least one mRNA transcript, it is classified as mRNA (or coding),
- otherwise if it includes at least one lncRNA transcript, it is classified as lncRNA (or long non-coding),
- otherwise it is classified as uncharacterized (or other).

#### Transcript QC metrics

Several measures are used to assess transcript quality, among which intron canonicity and transcript 5′ and 3′ completions, that we describe below.

##### Intron canonicity

For a given annotation, the first (donor side) and last (acceptor side) dinucleotide of each intron is extracted and checked for canonicity. An intron is considered canonical if the pair of its donor and acceptor dinucleotides corresponds to one of these pairs: (GT, AG), (GC, AG) or (AT,AC).

##### Transcript 5’ completion

Annotated Transcription Start Site (TSS) positions are compared with the ones of the GENE-SWitCH consensus ATAC-seq peaks obtained across all 84 GENE-SWitCH samples and available at the ENA using accessions ERZ10183160 and ERZ10454151. We defined the transcript 5’ completion as the percentage of TSS that overlap an ATAC-seq peak.

##### Transcript 3’ completion

Potential polyadenylation (polyA) signals were first localized in each genome with the seq2profile.pl and scanMotifGenomeWide.pl tools from the HOMER package (http://homer.ucsd.edu/homer/) using the ATTAAA and AATAAA consensus hexamers. Resulting positions were compared with the ones of the annotated Transcription Termination Sites (TTS). The percentage of non redundant TTS less distant than 50 bp from a polyA site was then computed to estimate the 3’ completion of the annotated transcripts.

#### TAGENS gene orthology with human

To infer orthology relationships, we adopted a projection-based approach. For a given livestock species, all its TAGENS transcripts (138,272 in chicken and 197,396 in pig) were projected to the human GRCh38 genome using:

- the UCSC pslMap program (Kent utils version 370, https://github.com/ENCODE-DCC/kentUtils),
- the UCSC pre-computed chain alignment between GRCh38 and the livestock genome assembly of interest (see above, called hg38To$spversion.over.chain.gz, human genome including haplotypes, https://hgdownload.soe.ucsc.edu/downloads.html),
- The psl format version of the TAGENS annotation.

This was done using the following command line:

pslMap -chainMapFile -mapInfo=mapInfo.file $species.annot.psl hg38To$spversion.over.chain.gz $species.to.hg38.psl

At the end of this process, each TAGENS transcript was associated to several alignments/hits (sets of alignment blocks), and in order to define the best one, we used the following standard alignment scoring scheme:

score(alignment) = matches + repMatches - misMatches - qNumInsert - tNumInsert

where:

- matches = Number of bp that match that aren't repeats

- misMatches = Number of bp that don't match

- repMatches = Number of bp that match & are part of repeats

- qNumInsert = Number of inserts in query

- tNumInsert = Number of inserts in target

In case a given transcript aligned to different places with the same score, the alignment with more blocks was picked, and in case of equality, the first alignment was picked.

Each TAGENS transcript’s best alignment on human was then selected and compared to human GRCh38 ENSEMBL v102 transcripts. In case a TAGENS transcript’s best alignment on human hits several human transcripts, the human transcript with the longest overlap was chosen along with its associated gene. Only exons with an overlap on the same strand were considered for that comparison (using the bedtools intersect command). In addition, each TAGENS gene was assigned the list of all overlapping ENSEMBL genes from the same species. Here also, an exonic overlap of at least one bp in the same orientation was required to consider two genes to overlap.

This way, we inferred orthology relationships by assigning to each TAGENS gene from species sp the two following pieces of information:

- A list of ENSEMBL sp genes
- A list of ENSEMBL human (hs) genes

Since ENSEMBL Biomart (https://www.ensembl.org/info/data/biomart/index.html, v102) provides known hs-sp gene orthology relationships for ENSEMBL protein coding genes, it was possible to use this subset as a ‘ground truth’ to estimate the sensitivity and the precision of our orthology inference process (that was applied to all TAGENS genes).

To compute the sensitivity, we looked for livestock TAGENS genes that colocalized with ENSEMBL genes that themselves have 1-to-1 orthologous genes in human. There were 12,856 chicken and 16,844 pig such TAGENS genes. For each gene belonging to this set, we looked whether the known human ortholog was included in the list of human genes hit by the projection of the TAGENS gene in human. As there were 11,206 chicken and 14,381 pig such TAGENS genes, and dividing this last number by the first one, we obtained a sensitivity of $87.2\%$ for chicken and $85.4\%$ for pig.

To compute the precision, we looked for human genes with a known 1-to-1 orthologous gene in livestock. There were 14,603 and 18,584 such human genes for chicken and pig respectively. For each human gene belonging to this set and with livestock ENSEMBL gene L that was 1-to-1 orthologous to it, we looked whether this human gene was hit by the projection of a TAGENS gene colocalizing with ENSEMBL gene L. As there were 12,582 and 16,934 such human genes, and dividing this last number by the first one, we obtained a precision of $86.2\%$ for chicken and $91.1\%$ for pig.

To infer lncRNA gene orthology relationships between livestock and human, we then extracted all possible triplets of consecutive genes (irrespective of strand) of the form (coding gene, lnc gene, coding gene) in the two livestock species and in human, and derived hs-sp lnc gene orthology relationships as described in Figure 5.

**Figure 3.**
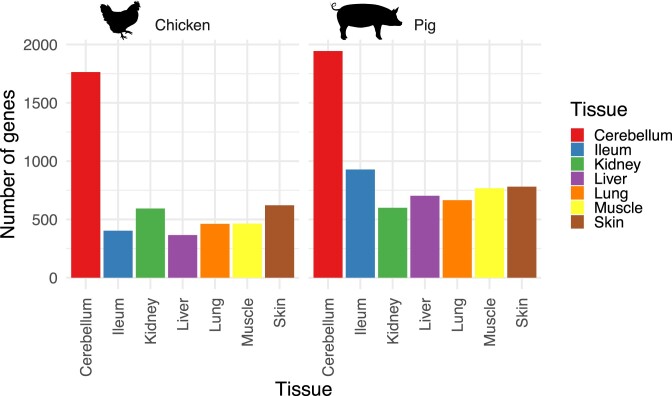
Number of tissue specific reference genes in each tissue for chicken (left) and pig (right). Tissue specific genes are defined as those with a tau index above 0.9 (Materials and Methods).

**Figure 4.**
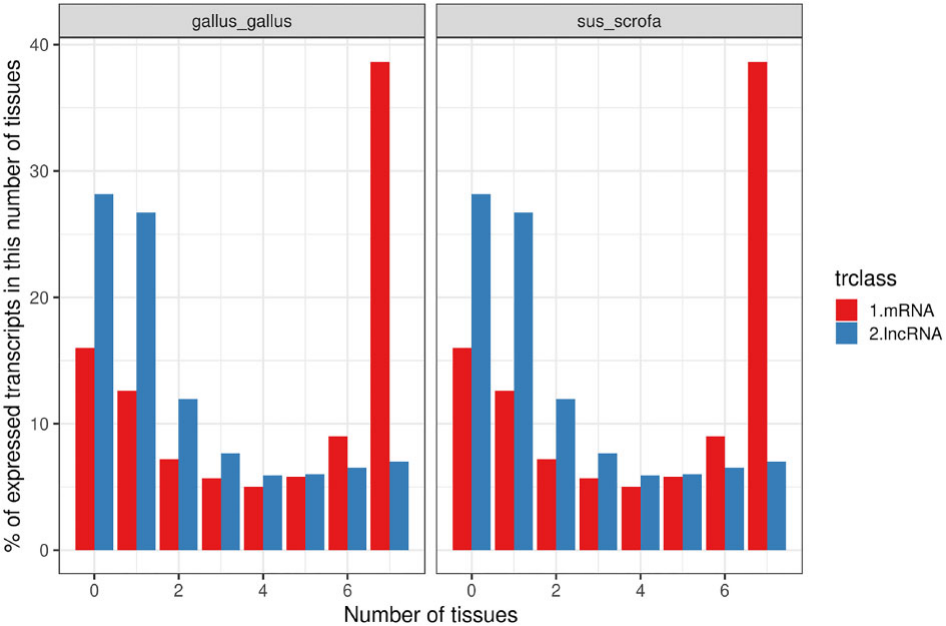
Percentage of mRNA and lncRNA transcripts expressed in 0, 1, 2, ..., 7 tissues.

**Figure 5.**
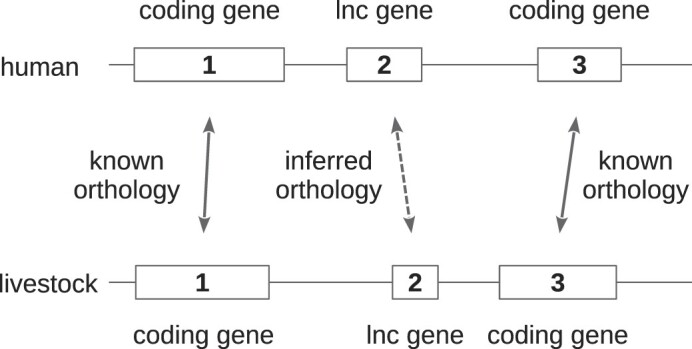
A simple synteny approach to infer orthology relationships between human and livestock long non-coding genes.

#### Transcript expression breadth

The expression breadth of a transcript corresponds to the number of tissues where the transcript is expressed. A transcript is considered expressed in a tissue if its TPM is above 0.1 in at least 4 out of the 12 (3 developmental stages and 4 animals) samples of the tissue.

#### Chicken annotation from TAGENS and the Rennes Atlas

A chicken TAGENS transcript is considered to be found in the Rennes Atlas if: (i) it is monoexonic and strandedly overlaps a monoexonic transcript from the Rennes Atlas or (ii) it is spliced and shares at least one intron with a transcript from the Rennes Atlas. A chicken TAGENS gene is considered to be found in the Rennes Atlas if one of its transcript is found in the Rennes Atlas.

#### Supplementary information

Additional data about this study, including a metadata summary table, the output TAGISO annotation, and output tables and scripts to generate this article’s figures, are provided as Supplementary Data Files at https://doi.org/10.57745/3UGLXW and on the project’s website https://www.fragencode.org/tagada.html. Additional scripts matrix_to_dist.R, ggheatmap.R, and comptr are included in the Docker image and available at https://github.com/sdjebali/.

## Results

To test the pipeline in a realistic use case, TAGADA was applied to improve the ENSEMBL reference annotation of the chicken and pig genomes using the Illumina RNA-seq data from the GENE-SWitCH project (Materials and Methods). A total of 84 FASTQ files were processed per species (4 animals, 7 tissues, and 3 developmental stages, Table 1).

### Gene expression profiling: reference annotation

Hierarchical clustering of the individual samples based on their ENSEMBL gene expression profiles showed that all bioreplicates of a given (tissue, stage) combination clustered together (Figure 2). Some tissues like cerebellum, liver, and kidney, consistently had all their samples clustering together (by stage), while others did not. We noticed that in pig, earlier developmental stage samples clustered altogether for muscle and skin as well as for ileum and lung, illustrating that expression programs can be mainly driven by either tissue or developmental stage.

In order to further assess the relevance of the reference gene quantification provided by TAGADA, we computed sets of tissue-specific genes using the tau index, which has been recognized as a robust measure of tissue-specificity (31) (Supplementary Figure S6). Requiring a tau value above 0.9 for calling tissue-specific genes, we found between 366 and 1,944 tissue-specific genes per tissue (Figure 3, Materials and Methods and Supplementary data file 4), with cerebellum being the tissue with the highest number of tissue specific genes in both species. Subsequent gene ontology (GO) enrichment analyses found relevant terms for the corresponding tissues, with for instance genes involved in axon guidance in cerebellum and muscle contraction in muscle (Supplementary Figure S2 and Materials and Methods). Overall, these results confirmed the relevance of the TAGADA quantification results.

### A novel genome annotation

Using the ENSEMBL reference annotation, TAGADA also produced a novel gene annotation herein called the TAGENS annotation (Supplementary Figure S4), with about three times more transcripts than in the reference (138,272 and 197,396, compared to 39,288 and 63,041 in the reference, for chicken and pig respectively). Importantly, about $70\%$ of those transcripts either exactly correspond to or are alternative isoforms of reference genes, while the other $30\%$ mainly characterize novel gene loci (Supplementary Figure S1 and Materials and Methods). TAGADA annotates between 1.4 and 1.6 time more genes than in the reference (34,712 and 51,171 TAGENS genes, compared to 24,356 and 31,908 in the reference, for chicken and pig respectively, Table 2). Those genes are almost equally divided into coding and long non-coding (lnc) genes (about $40\%$ each for the two species), while the remaining genes could neither be classified as coding nor as lnc and could correspond to small non-coding genes or to pseudogenes (Supplementary Figure S3 and Materials and Methods). In addition, for each TAGENS transcript, information of transcript id, gene id, transcript and gene coding status and transcript and gene novelty status (in terms of known/unknown) is provided in supplementary data file 2.

**Table 2.** Number of genes and transcripts included in the ENSEMBL, TAGENS, ISOSEQ and TAGISO gene annotations, along with the percentage of them that are expressed at TPM ≥0.1 in ≥2 samples

		Number of genes		Number of transcripts	
Species	Annotation	Total	Expressed	Total	Expressed
Chicken	ENSEMBL	24,356	21,102	39,288	33,233
	TAGENS	34,712	31,040	138,272	124,874
	ISOSEQ	22,473	22,118	139,658	128,859
	TAGISO	33,932	32,818	221,675	202,598
Pig	ENSEMBL	31,908	24,604	63,041	51,056
	TAGENS	51,171	42,595	197,396	171,103
	ISOSEQ	25,569	24,930	159,615	143,974
	TAGISO	46,717	44,408	268,634	239,759

A schematic overview of how these annotations were used or generated by TAGADA is provided in Supplementary Figure S4.

To validate the relevance of this novel TAGENS annotation, we performed two types of quality assessment. First, we controlled for a few expected features of the predicted transcript structures (Materials and Methods):

- the proportion of mono-exonic transcripts is not too high, and is lower than in the reference ($3.7\%$ and $4.6\%$ for chicken and pig, compared to $7.4\%$ and $8.6\%$ in ENSEMBL), which could partly be due to the long RNA selection process of the GENE-SWitCH RNA-seq protocol,
- the proportion of introns with canonical splice sites is slightly higher for TAGENS than for the reference ($99.5\%$ and $98.9\%$ compared to $98.3\%$ and $97.1\%$, for chicken and pig respectively),
- transcript completion is globally similar to that of the reference. On the one hand, TAGENS transcripts seem to be less complete at their 3′ end: $34.7\%$ (chicken) and $32.5\%$ (pig) of the TAGENS transcripts’ last 30 bp contain a polyadenylation signal, compared to $43.0\%$ and $42.8\%$ for the reference respectively. On the other hand, 5’ completion seems more accurate: $73.9\%$ (chicken) and $63.6\%$ (pig) of the distinct TAGENS TSS (Transcription Start Sites) are supported by GENE-SWitCH ATAC-seq peaks, as compared to $67.0\%$ and $64.0\%$ for the reference respectively.

Second, we checked whether novel TAGENS coding genes could be supported by human orthologs. Out of 980 chicken and 845 pig novel coding genes, a single human ortholog could respectively be found for 123 and 148 of them using a ”reciprocal-best-hit” sequence similarity approach (Materials and Methods). By further requiring that the TAGENS gene does not overlap any reference exon and that the human ortholog is coding and has no known ortholog in the livestock species, we were left with 85 chicken and 58 pig genes. Each of them had its respective one-to-one candidate ortholog in human. Interestingly enough, when looking for those genes in the most recent release of the ENSEMBL annotation upon writing this article (v108), we found that a large majority of those genes (77 and 50) were annotated by ENSEMBL since then. While part of this overlap is due to the GENE-SWitCH data being integrated into the v108 ENSEMBL annotation built, these results show that TAGADA can be used to identify new coding genes, even in relatively well annotated animal genomes.

Since non-extended reference transcripts, including the ones that might not be detected in the input samples, are systematically integrated in the novel annotation, some of them might get low or null expression values. By defining a threshold of 0.1 TPM in at least two samples for a gene or transcript to be considered as expressed, we found that most genes and transcripts from the novel annotation were expressed, which was already the case for the reference annotation even though to a lesser extent (Table 2).

### Long non-coding RNAs

Using FEELnc, the TAGADA pipeline classified $26.2\%$ (36,245 out of 138,272) transcripts for chicken and $22.6\%$ (44,625 out of 197,396) transcripts for pig, as lncRNAs. As a control, considering reference transcripts with a lncRNA biotype, we found that $88.0\%$ of the chicken reference lncRNAs (7,429 out of 8,446) and $89.2\%$ of the pig reference lncRNAs (8,008 out of 8,977) were also classified as lncRNAs by FEELnc. The remainder could not be confidently classified ($8.4\%$ for chicken and $8.0\%$ for pig) or were classified as mRNAs ($3.6\%$ for chicken and $2.7\%$ for pig). Most FEELnc lncRNA transcripts were intergenic ($70.9\%$ for chicken and $57.3\%$ for pig). Within the intergenic class, we found more transcripts antisense to their closest mRNA transcript and in divergent orientation (Supplementary Table S2). This predominant configuration could be explained by lncRNAs that are generated by bidirectional promoters of coding genes. Within the genic class, most transcripts were intronic and antisense to the gene they were located in ($58.9\%$ for chicken and $57.7\%$ for pig).

In total, considering both FEELnc and ENSEMBL biotypes (giving the priority to mRNA over lncRNA in case of conflict, Materials and Methods), we obtained 41,026 chicken and 50,946 pig TAGENS lncRNA transcripts. As expected and already observed in other studies (32), TAGENS lncRNAs were smaller: 1,792 bp and 2,180 bp median lncRNA transcript length in chicken and pig respectively, compared to 2,069 bp and 3,114 bp for mRNA transcripts (*P*-value < 2.2 × 10^−16^, Wilcoxon test). They also included less exons: 3 and 2 median number of exons per lncRNA transcript in chicken and pig respectively, compared to 8 and 9 for mRNA transcripts (*P*-value < 2.2 × 10^−16^, Wilcoxon test). LncRNA transcripts were also less broadly expressed than coding transcripts (Figure 4, Materials and Methods, Supplementary data file 5, (32)).

A recent study has produced a quite exhaustive atlas of chicken genes, herein called the Rennes Atlas, by gathering information from diverse databases, such as ENSEMBL, FR-AgENCODE (INRA), NONCODE, ALDB, NCBI and RefSeq (33). The Rennes Atlas comprises a total of 45,615 genes, of which 25,082 ($55.0\%$) are long non-coding and 17,921 ($39.3\%$) are coding. The source of these genes by biotype is provided in Supplementary Figure S5.

The Rennes Atlas includes 72,306 transcripts, with 39,643 ($54.8\%$) identified as lncRNAs and 29,737 ($41.1\%$) as coding (Materials and Methods). In total, 20,257 out of 41,026 ($49.4\%$) TAGENS lncRNA transcripts and 6,986 out of 14,872 ($47.0\%$) TAGENS lncRNA genes were found in the Rennes Atlas (Materials and Methods). Focusing on the TAGENS unknown lncRNA transcripts and genes the proportions found in the Rennes Atlas reached $36.6\%$ (11,991 out of 32,760) and $24.4\%$ (2,551 out of 10,437), respectively. Importantly, the majority of these TAGENS unknown lncRNA transcripts/genes that were found in the Rennes Atlas hit a transcript/gene that is not classified as an mRNA in the Rennes Atlas, with a high percentage of $98.1\%$ (11,760 out of 11,991) for transcripts and $93.1\%$ (2,374 out of 2,551) for genes. These results emphasize the consistency between both studies.

An important amount of TAGENS genes (14,972 in chicken and 22,691 in pig) do not have any transcript exactly matching a reference transcript. We refer to this gene category as unknown. Most of them (10,437 in chicken and 16,661 in pig) are long non-coding. In order to investigate their relevance, we checked whether some of them could feature some evolutionary conservation by looking for possible orthologs in human. Given that lncRNAs are usually poorly conserved at the sequence level between vertebrates (32), we used a synteny approach to infer orthology relationship between lncRNAs (34). In brief, we looked for conserved triplets of genes between species with two coding genes flanking the lncRNA gene (Materials and Methods, Figure 5). While this approach does not guarantee the discovery of all orthologous lncRNA pairs or definitively establish orthology relationships between them, it has demonstrated reliability in previous studies (33–35). Thus, we identified 1,370 and 1,841 such triplets involving unknown lncRNA genes in chicken and pig respectively. Comparison with a set of 2,712 human triplets revealed 64 chicken and 134 pig lncRNA genes with a predicted orthology in human, of which 2 were common to both species.

### Improving a long-read based annotation with short reads

The structural annotation of transcripts expressed in a given sample is more easily and confidently performed when long-read RNA-seq data are available, for instance from Oxford Nanopore or PacBio technologies (36). As the transcriptomes of the 21 combinations of 7 tissues and 3 developmental stages of the GENE-SWitCH project were also profiled by PacBio Iso-Seq for the two species, we investigated whether TAGADA could improve a long-read derived gene annotation with Illumina short reads.

Toward this goal, we ran the same analysis as above but instead of using the ENSEMBL annotation as a reference, we used the annotation that was previously generated by processing the Iso-Seq long reads with the nf-core/isoseq pipeline (37). We refer to this input annotation as the IsoSeq annotation, and to the TAGADA annotation obtained by integrating the Illumina short reads to this reference annotation, as the TAGISO annotation (as opposed to TAGENS for the TAGADA run described above, that used the ENSEMBL annotation as input, Supplementary Figure S4 and Supplementary data file 3). Resulting numbers of genes and transcripts are displayed in Table 2.

As expected (since short and long read data come from the same samples), the resulting TAGISO annotation displayed a better agreement with the IsoSeq annotation than the TAGENS annotation did with the ENSEMBL annotation. For instance, the percentage of expressed reference genes for chicken and pig increased from 81% and 61% with TAGENS to 92% and 91% for TAGISO. The percentage of spliced TAGENS transcripts that exactly matched the reference transcripts for chicken and pig also increased from 54% and 29% for TAGENS to 76% and 51% for TAGISO. These improvements, due to the fact that the Iso-Seq and NovaSeq experiments were mostly performed on the same samples, showed the consistency of the TAGADA output in this context. The fact that about $98\%$ of the Iso-seq genes were considered as expressed using short read quantification further supports this consistency (Table 2).

The number of TAGISO and TAGENS genes were quite similar (33,932 and 34,712 for chicken, and 46,717 and 51,171 for pig), but corresponded to 1.5 and 1.8 times more genes than in the IsoSeq reference annotation, showing the ability of TAGADA to detect genes previously undetected by IsoSeq. The number of TAGISO transcripts, however, was higher than the number of TAGENS transcripts (about 222K for chicken and 269K for pigs, compared to 138K and 197K in TAGENS, respectively). It was also higher than the number of IsoSeq transcripts (about 140K for chicken and 160K for pig). This suggests that TAGADA can successfully use short reads to detect transcripts that were missed when using long reads only, leveraging the complementarity between these technologies.

While the majority of TAGISO transcripts corresponded to IsoSeq transcripts, there were also many TAGISO spliced transcripts that extended IsoSeq transcripts by adding at least one exon in the 5′ and/or in the 3′ direction (about 40K such transcripts in each species). Among those, we saw that there were less transcripts extending the IsoSeq transcripts in 3′ than in 5′ (Supplementary Table S3, Materials and Methods). We hypothesized that although the IsoSeq transcripts are supposed to be complete in 5′, they are also known to be much more complete in 3′ (by definition), which is also shown by the very high percentage of IsoSeq transcript ends harbouring a polyA site ($76.4\%$ and $75.4\%$ for chicken and pig, Materials and Methods).

## Discussion

Genome annotation is a crucial step in understanding the genetic makeup of a species. With the continuous generation of new transcriptome data from research projects worldwide, it is essential to integrate these data into existing annotations in a consistent and reproducible manner. Beyond the general need for the community to update and maintain reference annotations, a more specific challenge is for individual transcriptome projects to provide an improved and customized version of a reference genome annotation. In this particular context, the goal is to use the project’s RNA-seq data in order to identify and assess the expression of novel genes and transcripts of interest under specific biological conditions.

In some cases, waiting for the release of a reference annotation built with the data of interest might appear as a viable option, leveraging the established expertise of annotation groups. However, this strategy is not always feasible due to several compelling reasons, including, for instance, the lack of control and visibility on the release schedule, the integrated data, and the integration methods. Due to the lack of open-source code and practical reproducibility of reference annotation pipelines, relying solely on the resulting annotations inhibits the capacity to tailor the analysis process to the specific requirements of the project, which can be a major issue.

The TAGADA pipeline presented in this study provides a comprehensive solution to address this need. By applying TAGADA to improve the ENSEMBL reference annotation of the chicken and pig genomes within the GENE-SWitCH RNA-seq project, we demonstrated the effectiveness of TAGADA in enhancing genome annotations.

Due to the absence of a species with a completely and perfectly annotated genome, assessing the validity of a genome annotation is a complex task. In this study we presented various analyses to support the relevance of TAGADA’s output. First, gene expression profiling of reference genes generated consistent hierarchical clustering of the samples in both species, providing confidence in the quantification results. Furthermore, Gene Ontology analyses on tissue-specific genes supported the biological relevance of the TAGADA quantification results. Next, TAGADA generated a novel genome annotation for the chicken and pig genomes, drastically increasing the number of genes and transcripts compared to the reference annotation. Quality control metrics on gene and transcript features confirmed the validity of the novel annotation, and orthology analyses from chicken or pig to human showed that TAGADA can identify new coding genes, even in relatively well-annotated animal genomes.

The identification of long non-coding RNAs is another important aspect of the transcriptome analysis that can no longer be overlooked. In this study, about half of the genes from the novel annotations were classified as non-coding. Interestingly, orthology analyses with human genes revealed the presence of synteny-conserved lncRNAs, representing promising candidates for further investigation and functional characterization. Last, by integrating Illumina NovaSeq short reads with an annotation derived from PacBio Iso-Seq long reads, we demonstrated the versatility of TAGADA and showcased how it can harness the complementarity between sequencing technologies.

In summary, TAGADA provides a comprehensive and powerful solution for improving genome annotations. In particular, its ability to generate a novel annotation from a large set of samples and to characterize lncRNAs makes it a unique and valuable tool for the scientific community. The results obtained in this study demonstrate the effectiveness of TAGADA in extending the number of annotated coding and non-coding genes and transcripts, allowing for a better understanding of complex animal genomes.

## Supplementary Material

lqad089_Supplemental_File

## Data Availability

TAGADA's source code is available in Zenodo at https://doi.org/10.5281/zenodo.8322250. Additional data files are available in the INRAE Omics Dataverse at https://doi.org/10.57745/3UGLXW. General information about the TAGADA pipeline is available on the FR-AgENCODE website at https://www.fragencode.org/tagada.html.
