# Supplementary material for: TAGADA: a scalable pipeline to improve genome annotations with RNA-seq data
Source: NAR Genom Bioinform. 2023 Oct 16;5(4):lqad089. doi: 10.1093/nargab/lqad089 (PMC10578202; doi:10.1093/nargab/lqad089)
Supplement: lqad089_Supplemental_File [file lqad089_supplemental_file.pdf]

*TAGADA: a scalable pipeline to improve genome annotations with RNA-seq data*

# Supplementary file 1: Supplementary figures and tables

# List of Figures

S1 **TAGENS transcript positional class with respect to ENSEMBL reference annotation**. . . 3
S2 **GO term enrichment analysis for chicken and pig tissue-specific genes.** From top to bottom are provided enriched GO terms from the biological process (BP) ontology, with a $10^{-5}$ significance threshold . . . . . . . . . . . . . . . . . . . . . . . . . . . . . . . . . . . . . . 4
S3 **TAGENS transcript and gene coding and novelty classification**. . . . . . . . . . . . . . . 5
S4 **Overview of the various transcriptome analyses considered in the study. Input and output annotations are indicated for each annotation, along with the pipeline name and the transcriptome data that was processed. The Isoseq annotation was generated in the context of the GENE-SWitCH project and retrieved from the ENA (accessions ERZ15610616 and ERZ15610622).** . . . . . . . . . . . . . . . . . . . . . . . . . . . . . 6
S5 **Source of Rennes chicken atlas genes per gene biotype.** . . . . . . . . . . . . . . . . . . . 7
S6 **Distribution of tau values for genes of the Ensembl annotation** . . . . . . . . . . . . . . 8

# List of Tables

S1 **Popular RNA-seq data analysis pipelines**. WML: Workflow Management Language. SM: Snakemake. NF: Nextflow. Ref. only: only the reference annotation is quantified. DEA: Differential Expression Analysis. scRNA-seq: single-cell RNA-seq analysis. (*)TPM only, quantification is performed on each novel annotation separately. (**)TPM and raw read counts, quantification is performed on the same novel annotation. . . . . . . . . . . . . . . . . . . . . . 9
S2 `FEELnc` **lncRNA transcript classification.** . . . . . . . . . . . . . . . . . . . . . . . . . . . . . 9
S3 `TAGISO` **transcripts extending** `Iso-Seq` **transcripts.** . . . . . . . . . . . . . . . . . . 10

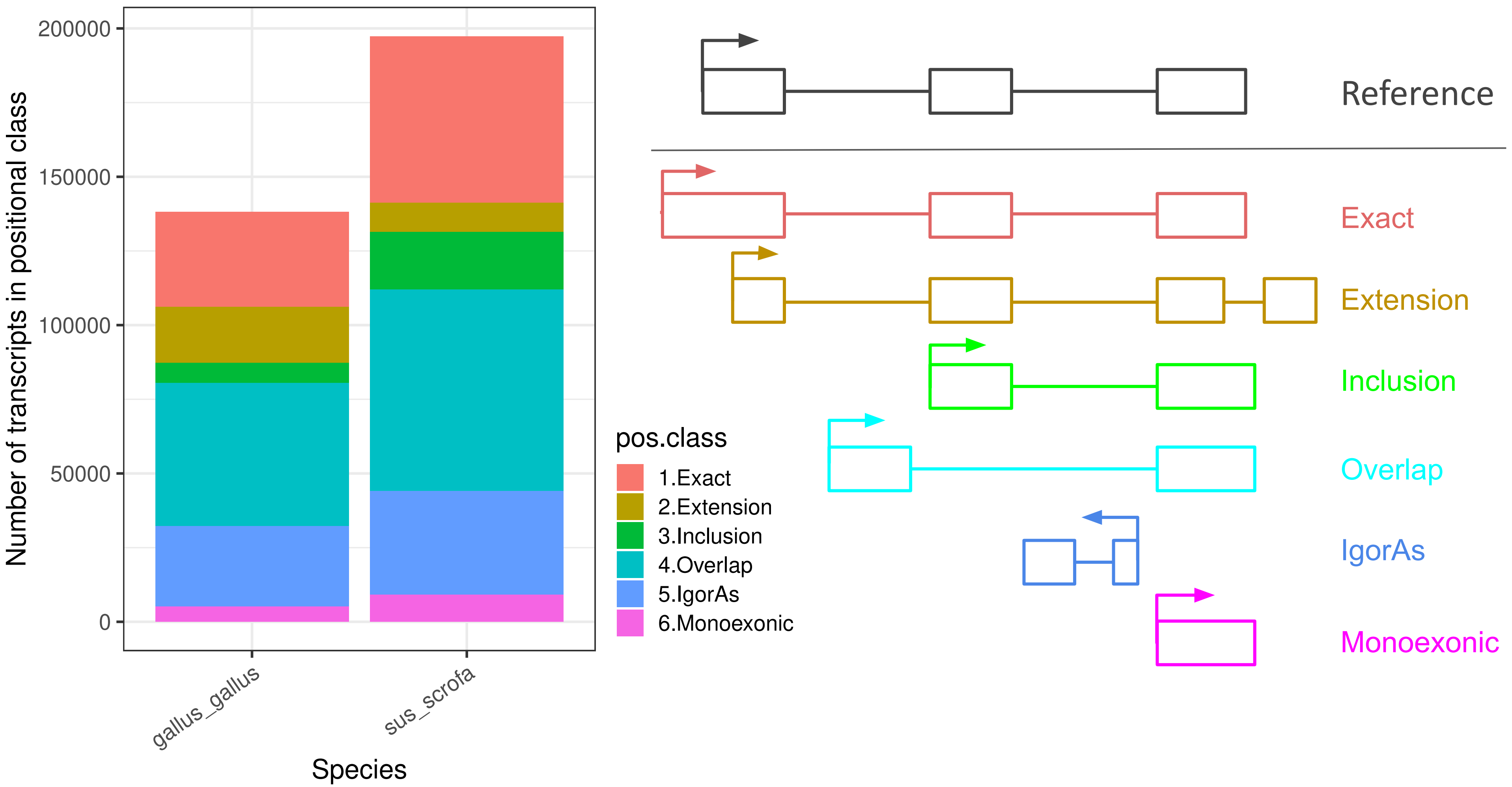


Figure S1: **TAGENS transcript positional class with respect to ENSEMBL reference annotation.**

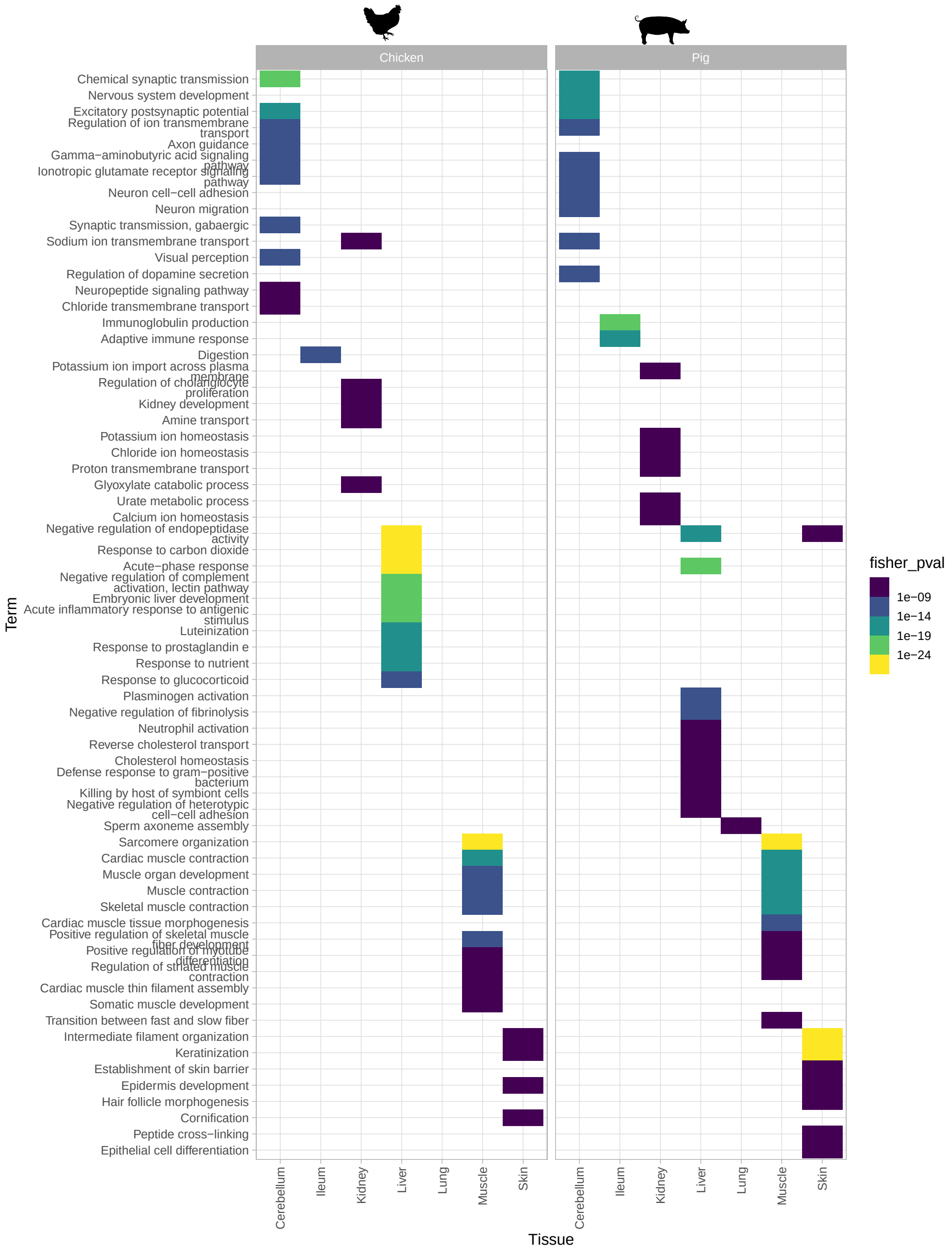


Figure S2: **GO term enrichment analysis for chicken and pig tissue-specific genes.** From top to bottom are provided enriched GO terms from the biological process (BP) ontology, with a $10^{-5}$ significance threshold

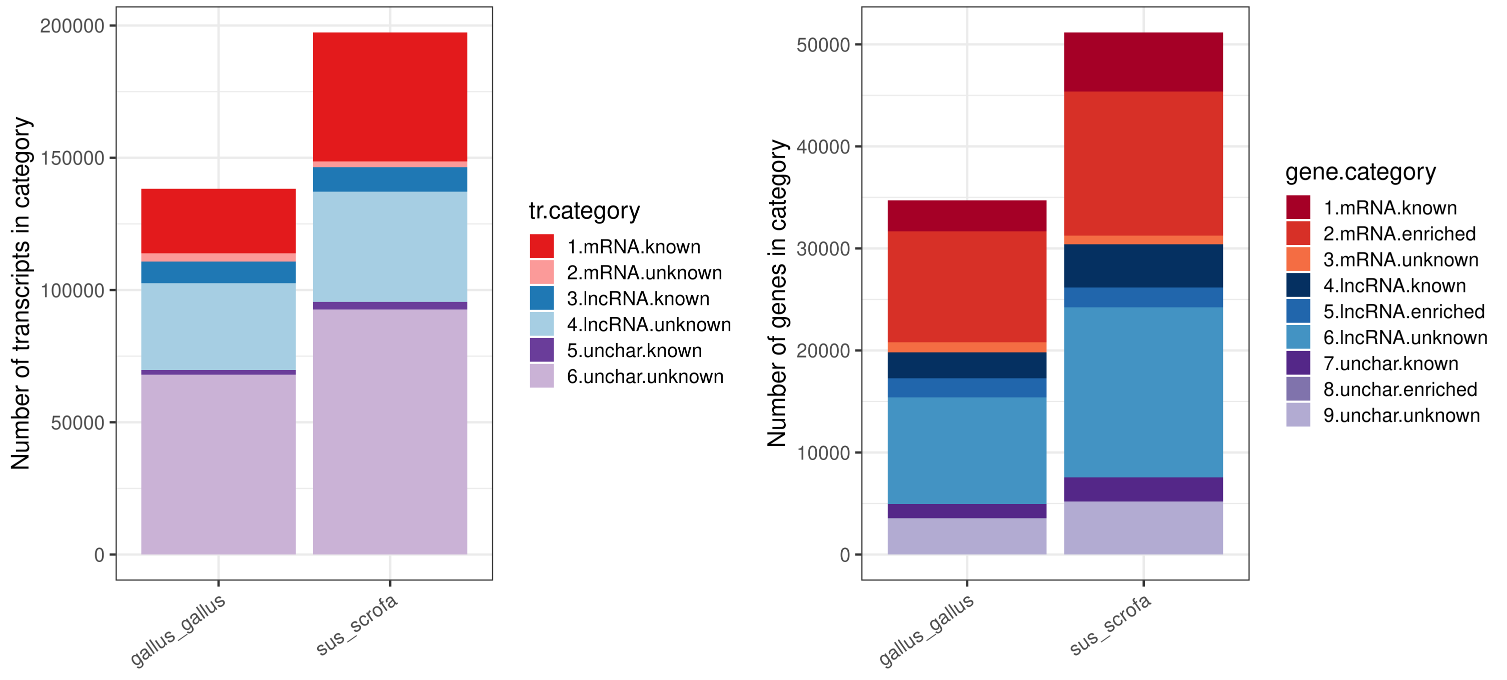


Figure S3: **TAGENS transcript and gene coding and novelty classification.**

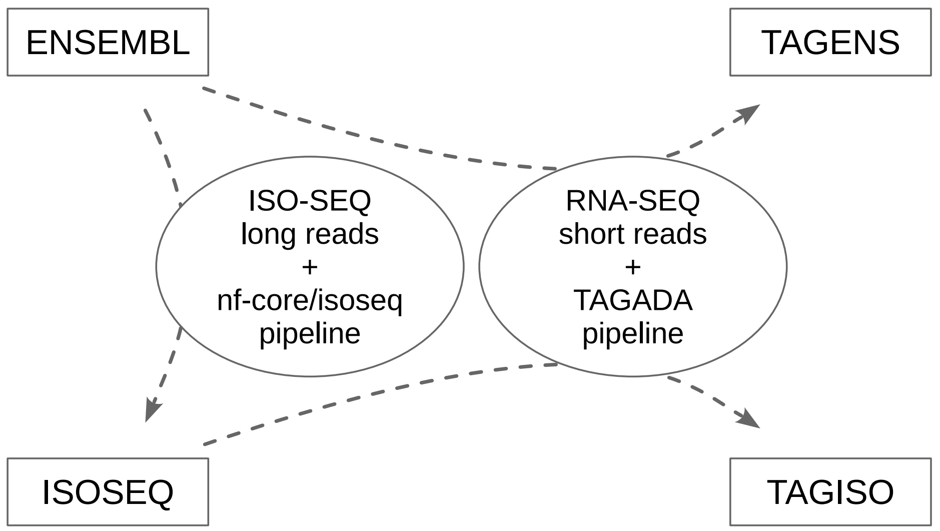


Figure S4: **Overview of the various transcriptome analyses considered in the study. Input and output annotations are indicated for each annotation, along with the pipeline name and the transcriptome data that was processed. The Isoseq annotation was generated in the context of the GENE-SWitCH project and retrieved from the ENA (accessions ERZ15610616 and ERZ15610622).**

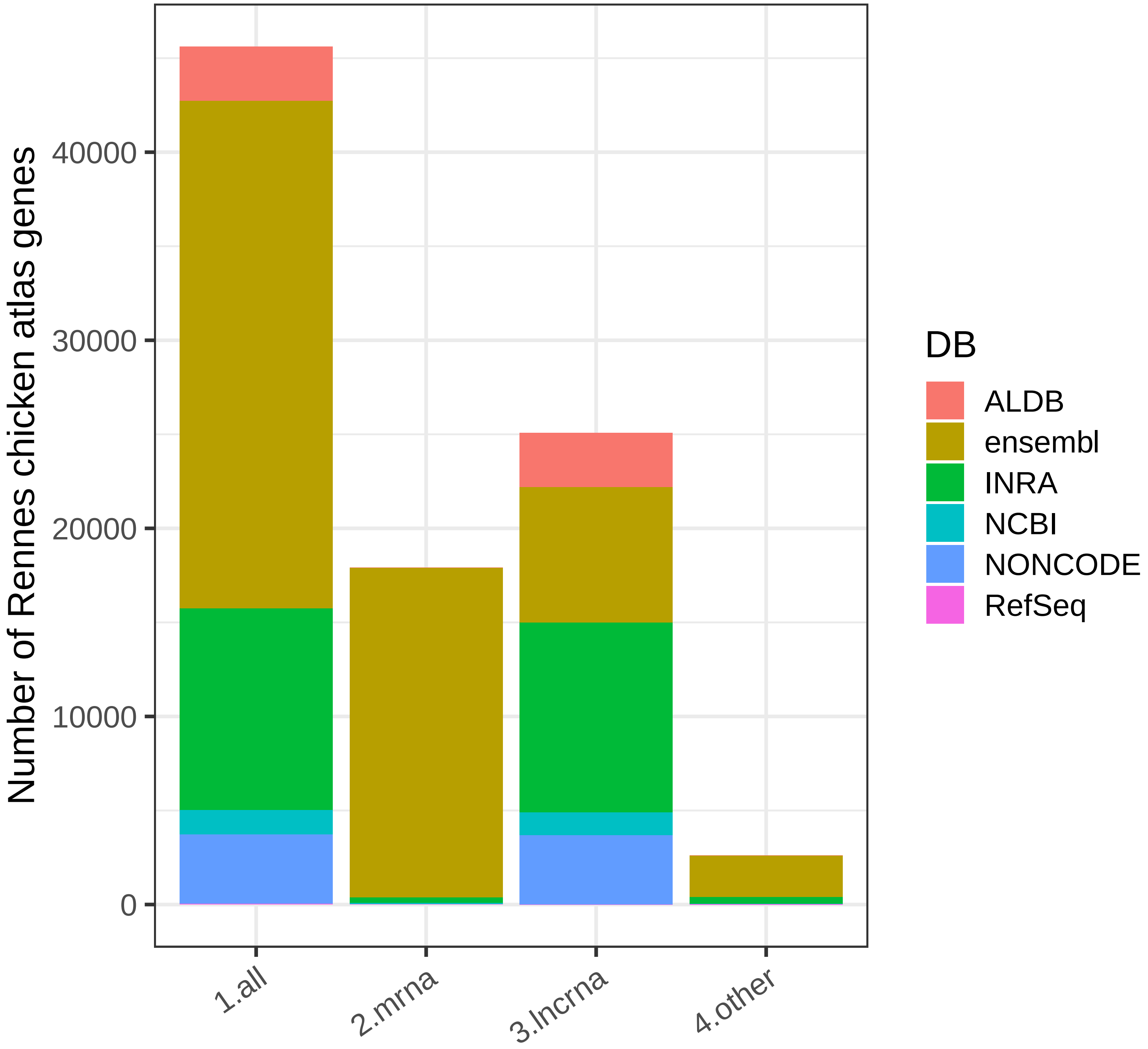


Figure S5: **Source of Rennes chicken atlas genes per gene biotype.**

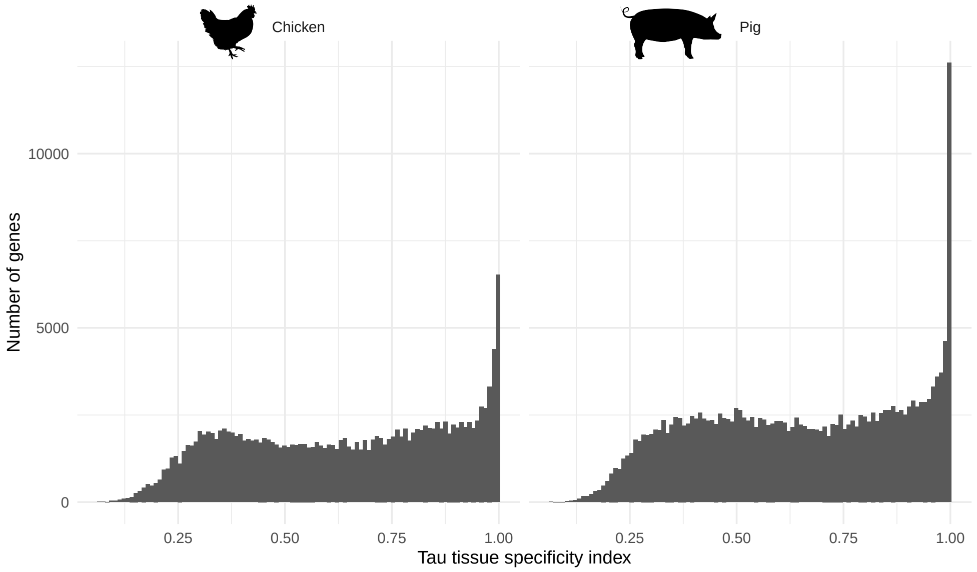


Figure S6: **Distribution of tau values for genes of the Ensembl annotation**

| Pipeline name | WML | Software environment | Novel annotation | Expression quantification | lncRNA detection | Selected features |
|---|---|---|---|---|---|---|
| hppRNA[1] | SM | No | No | Ref. only | iSeeRNA | DEA, gene fusion detection |
| VIPER[2] | SM | Conda | No | Ref. only | None | DEA |
| ARMOR[3] | SM | Conda | No | Ref. only | None | DEA |
| Pipeliner[4] | NF | No | No | Ref. only | None | scRNA-seq |
| nf-core /rnaseq[5] | NF | Docker, Singularity | Disparate annotations | Ref. and novel(*) | None | Reference framework |
| RASflow[6] | SM | Docker | No | Ref. only | None | DEA |
| GEMmaker[7] | NF | Docker, Singularity | No | Ref. only | None | Resource efficient |
| transXpress[8] | SM | Conda | Integrated annotation | Nov. only | None | de novo assembly |
| TAGADA | NF | Docker, Singularity | Integrated annotation | Ref. and novel(**) | FEELNc | Annotation improvement |

Table S1: **Popular RNA-seq data analysis pipelines**. WML: Workflow Management Language. SM: Snakemake. NF: Nextflow. Ref. only: only the reference annotation is quantified. DEA: Differential Expression Analysis. scRNA-seq: single-cell RNA-seq analysis. (*)TPM only, quantification is performed on each novel annotation separately. (**)TPM and raw read counts, quantification is performed on the same novel annotation.

| **Species** | **# lncRNA transcripts** | **Intergenic (IG)** | | **Genic** | |
|---|---|---|---|---|---|
| | | # | % of total | # | % of total |
| Chicken | 36,245 | 25,696 | **70.9** | 10,549 | 29.1 |
| Pig | 44,625 | 25,565 | **57.3** | 19,060 | 42.7 |

| **Species** | **# IG lncRNA transcripts** | **IG sense (S)** | | **IG antisense (AS)** | | **IG S upstream** | | **IG S downstream** | | **IG AS divergent** | | **IG AS convergent** | |
|---|---|---|---|---|---|---|---|---|---|---|---|---|---|
| | | # | % of IG | # | % of IG | # | % of IG S | # | % of IG S | # | % of IG AS | # | % of IG AS |
| Chicken | 25,696 | 11,425 | 44.5 | 14,271 | **55.5** | 5,674 | 49.7 | 5,751 | 50.3 | 9,187 | **64.4** | 5,084 | 35.6 |
| Pig | 25,565 | 10,733 | 42.0 | 14,832 | **58.0** | 5,178 | 48.2 | 5,555 | 51.8 | 10,094 | **68.1** | 4,738 | 31.9 |

| **Species** | **# genic lncRNA transcripts** | **Genic intronic AS** | | **Genic exonic AS** | | **Genic intronic S** | | **Genic exonic S** | |
|---|---|---|---|---|---|---|---|---|---|
| | | # | % of genic | # | % of genic | # | % of genic | # | % of genic |
| Chicken | 10,549 | 6,214 | **58.9** | 2,034 | 19.3 | 891 | 8.4 | 1,410 | 13.4 |
| Pig | 19,060 | 11,001 | **57.7** | 4,297 | 22.5 | 2,139 | 11.2 | 1,623 | 8.5 |

Table S2: `FEELnc` **lncRNA transcript classification.**

| Species | **Number of `TAGISO` transcripts extending an `Iso-Seq` transcript** | **Only in the 5' direction (% of total)** | **Only in the 3' direction (% of total)** | **In both the 5' and the 3' directions (% of total)** |
|---|---|---|---|---|
| Chicken | $41,746$ | $29,920$ ($71.7$) | $6,499$ ($15.6$) | $4,951$ ($11.9$) |
| Pig | $44,430$ | $30,232$ ($68.0$) | $7,610$ ($17.1$) | $6,181$ ($13.9$) |

Table S3: `TAGISO` **transcripts extending** `Iso-Seq` **transcripts.**

# References


[1] Dapeng Wang. hpprna - a snakemake-based handy parameter-free pipeline for rna-seq analysis of numerous samples. *Briefings in bioinformatics*, 19(4):622–626, 2018.

[2] MacIntosh Cornwell, Mahesh Vangala, Len Taing, Zachary Herbert, Johannes Köster, Bo Li, Hanfei Sun, Taiwen Li, Jian Zhang, Xintao Qiu, et al. Viper: Visualization pipeline for rna-seq, a snakemake workflow for efficient and complete rna-seq analysis. *BMC bioinformatics*, 19:1–14, 2018.

[3] Stephany Orjuela, Ruizhu Huang, Katharina M Hembach, Mark D Robinson, and Charlotte Soneson. Armor: An a utomated r eproducible mo dular workflow for preprocessing and differential analysis of r na-seq data. *G3: Genes, Genomes, Genetics*, 9(7):2089–2096, 2019.

[4] Anthony Federico, Tanya Karagiannis, Kritika Karri, Dileep Kishore, Yusuke Koga, Joshua D Campbell, and Stefano Monti. Pipeliner: a nextflow-based framework for the definition of sequencing data processing pipelines. *Frontiers in genetics*, 10:614, 2019.

[5] Philip A Ewels, Alexander Peltzer, Sven Fillinger, Harshil Patel, Johannes Alneberg, Andreas Wilm, Maxime Ulysse Garcia, Paolo Di Tommaso, and Sven Nahnsen. The nf-core framework for community-curated bioinformatics pipelines. *Nature biotechnology*, 38(3):276–278, 2020.

[6] Xiaokang Zhang and Inge Jonassen. Rasflow: an rna-seq analysis workflow with snakemake. *BMC bioinformatics*, 21(1):1–9, 2020.

[7] John A Hadish, Tyler D Biggs, Benjamin T Shealy, M Reed Bender, Coleman B McKnight, Connor Wytko, Melissa C Smith, F Alex Feltus, Loren Honaas, and Stephen P Ficklin. Gemmaker: process massive rna-seq datasets on heterogeneous computational infrastructure. *BMC bioinformatics*, 23(1):156, 2022.

[8] Timothy R Fallon, Tereza Čalounová, Martin Mokrejš, Jing-Ke Weng, and Tomáš Pluskal. transxpress: a snakemake pipeline for streamlined de novo transcriptome assembly and annotation. *BMC bioinformatics*, 24(1):1–11, 2023.
